# Trichotomy for Dynamical Systems in Banach Spaces

**DOI:** 10.1155/2013/793813

**Published:** 2013-09-29

**Authors:** Codruţa Stoica

**Affiliations:** Department of Mathematics and Computer Science, Aurel Vlaicu University of Arad, 2 Elena Drăgoi Str, 310330 Arad, Romania

## Abstract

We construct a framework for the study of dynamical systems that describe phenomena from physics and engineering in infinite dimensions and whose state evolution is set out by skew-evolution semiflows. Therefore, we introduce the concept of *ω*-trichotomy. Characterizations in a uniform setting are proved, using techniques from the domain of nonautonomous evolution equations with unbounded coefficients, and connections with the classic notion of trichotomy are given. The statements are sustained by several examples.

## 1. Introduction

The possibility of reducing the nonautonomous case in the study of associated evolution operators to the autonomous case of evolution semigroups on various Banach function spaces can be considered as an important way towards applications issued from the real world. Of great importance in the study the solution of differential equations is the approach by evolution families, as the techniques from the domain of non autonomous equations with unbounded coefficients in infinite dimensions can be extended in this direction.

Appropriate for the study of evolution equations in infinite dimensions are also the skew-evolution semiflows, introduced by us in [1], as generalizations for evolution operators and skew-product semiflows, and whose applicability is pointed out, for example, by Bento and Silva in [2] and by Hai in [3, 4]. Other asymptotic properties for skew-evolution semiflows are defined and characterized in [5, 6].

The techniques used in the investigation of exponential stability and exponential instability were generalized for the case of exponential dichotomy in [7, 8] and for the case of exponential trichotomy in [9, 10]. The main idea in the study of trichotomy, introduced for the finite dimensional case by Sacker and Sell in [11], as a natural generalization of the concept of dichotomy is to obtain, at any moment, a decomposition of the state space into three subspaces: a stable subspace, an instable one, and a third one, the central manifold. The aim of the present study is to emphasize the property of *ω*-trichotomy for skew-evolution semiflows in Banach spaces and to give several conditions in order to describe the behavior related to the third subspace.

## 2. Skew-Evolution Semiflows

Let (*X*, *d*) be a metric space, *V* a Banach space, and V* its topological dual. Let *ℬ*(*V*) be the space of all *V*-valued bounded operators defined on *V*. The norm of vectors on *V* and on V* and of operators on *ℬ*(*V*) is denoted by ||·||. Let us consider that *Y* = *X* × *V* and *T* = {(*t*, *t*_0_) ∈ ℝ_+_^2^ : *t* ≥ *t*_0_}. *I* is the identity operator.

**Definition 1**

A mapping *φ* : *T* × *X* → *X* is called *evolution semiflow* on *X* if the following properties are satisfied:

(es1) $$\begin{array}{c} \varphi \left(t,t,x\right)=x,\;\forall \left(t,x\right)\in {\mathbb{R}}_{+}\times X, \end{array}$$

(es2) φ(t,s,φ(s,t0,x))=φ(t,t0,x), ∀(t,s),(s,t0)∈T,             ∀x∈X.

**Definition 2**

A mapping Φ : *T* × *X* → *ℬ*(*V*) is called *evolution cocycle* over an evolution semiflow *φ* if it satisfies the following properties:

(ec1) $$\begin{array}{c} \Phi \left(t,t,x\right)=I,\;\forall t\geq 0,\;\forall x\in X, \end{array}$$

(ec2) Φ(t,s,φ(s,t0,x))Φ(s,t0,x)  =Φ(t,t0,x), ∀(t,s),(s,t0)∈T, ∀x∈X.

**Definition 3**

The mapping *C* : *T* × *Y* → *Y* defined by

(1) $$\begin{array}{c} C\left(t,s,x,v\right)=\left(\varphi \left(t,s,x\right),\Phi \left(t,s,x\right)v\right), \end{array}$$

where Φ is an evolution cocycle over an evolution semiflow *φ*, is called *skew-evolution semiflow* on *Y*.

**Example 4**

Let *f* : ℝ_+_ → ℝ_+_* be a decreasing function with the property that there exists lim⁡_*t*→*∞*_⁡*f*(*t*) = *a* > 0. We denote by *𝒞* = *𝒞*(ℝ_+_, ℝ_+_) the set of all continuous functions *x* : ℝ_+_ → ℝ_+_, endowed with the topology of uniform convergence on compact subsets of ℝ_+_, metrizable by means of the distance

(2) $$\begin{array}{c} d\left(x,y\right)=\sum_{n=1}^{\infty }\frac{1}{2^{n}}\frac{d_{n}\left(x,y\right)}{1+d_{n}\left(x,y\right)},\\ \text{where}\;d_{n}\left(x,y\right)=\underset{t\in \left[0,n\right]}{\sup}\left|x\left(t\right)-y\left(t\right)\right|. \end{array}$$

If *x* ∈ *𝒞*, then, for all *t* ∈ ℝ_+_, we denote that *x*_*t*_(*s*) = *x*(*t* + *s*), *x*_*t*_ ∈ *𝒞*. Let *X* be the closure in *𝒞* of the set {*f*_*t*_, *t* ∈ ℝ_+_}. It follows that (*X*, *d*) is a metric space, and the mapping *φ* : Δ × *X* → *X*, *φ*(*t*, *s*, *x*) = *𝓍*_*t*−*s*_ is an evolution semiflow on *X*.

We consider that *V* = ℝ^2^, with the norm ||*v*|| = |*v*_1_| + |*v*_2_|, *v* = (*v*_1_, *v*_2_) ∈ *V*. If *u* : ℝ_+_ → ℝ_+_*, then the mapping Φ_*u*_ : Δ × *X* → *ℬ*(*V*) defined by

(3) $$\begin{array}{c} \Phi_{u}\left(t,s,𝓍\right)v=\left(\frac{u\left(s\right)}{u\left(t\right)}e^{-\int_{s}^{t}𝓍(\tau -s)d\tau }v_{1},\frac{u\left(t\right)}{u\left(s\right)}e^{\int_{s}^{t}𝓍(\tau -s)d\tau }v_{2}\right) \end{array}$$

is an evolution cocycle over *φ*, and *C* = (*φ*, Φ_*u*_) is a skew-evolution semiflow.

A particular class of skew-evolution semiflows is emphasized in the following.

**Remark 5**

Let us consider a skew-evolution semiflow *C* = (*φ*, Φ) and a parameter *λ* ∈ ℝ. We define the mapping

(4) $$\begin{array}{c} \Phi_{\lambda }:T\times X\to ℬ\left(V\right),\;\Phi_{\lambda }\left(t,t_{0},x\right)=e^{-\lambda (t-t_{0})}\Phi \left(t,t_{0},x\right). \end{array}$$

We observe that *C*_*λ*_ = (*φ*, Φ_*λ*_) is also a skew-evolution semiflow, called *λ-shifted skew-evolution semiflow* on *Y*. We will call Φ_*λ*_ the *λ-shifted evolution cocycle*.

**Example 6**

Let *X* be the metric space defined in the first Example. We define the mapping *φ*_0_ : ℝ_+_ × *X* → *X*, *φ*_0_(*t*, *x*) = *x*_*t*_, where *x*_*t*_(*τ*) = *x*(*t* + *τ*), for all *τ* ≥ 0, which is a classic semiflow on *X*. Let us consider for every *x* ∈ *X* the parabolic system with Neumann's boundary conditions as follows:

(5) $$\begin{array}{c} \frac{\partial v}{\partial t}\left(t,y\right)=x\left(t\right)\frac{\partial^{2}v}{\partial y^{2}}\left(t,y\right),\;t>0,\;\;y\in \left(0,1\right),\\ v\left(0,y\right)=v_{0}\left(y\right),\;y\in \left(0,1\right),\\ \frac{\partial v}{\partial y}\left(t,0\right)=\frac{\partial v}{\partial y}\left(t,1\right)=0,\;t>0. \end{array}$$

Let *V* = *ℒ* ^2^(0,1) be a separable Hilbert space with the orthonormal basis {*e*_*n*_}_*n*∈*ℕ*_, *e*_0_ = 1, $e_{n}\left(y\right)=\sqrt{2}\cos n\pi y$, where *y* ∈ (0,1), *n* ∈ *ℕ*.

We denote that *D*(*A*) = {*v* ∈ *ℒ* ^2^(0,1), *v*(0) = *v*(1) = 0}, and we define the operator

(6) $$\begin{array}{c} A:D\left(A\right)\subset V\to V,\;Av=\frac{d^{2}v}{dy^{2}}, \end{array}$$

which generates a *𝒞*_0_-semigroup *S*, defined by

(7) $$\begin{array}{c} S\left(t\right)v=\sum_{n=0}^{\infty }e^{-n^{2}\pi^{2}t}\langle v,e_{n}\rangle e_{n}, \end{array}$$

where 〈·, ·〉 denotes the scalar product in *V*. For every *𝓍* ∈ *X*, let us define an operator *A*(*x*) : *D*(*A*) ⊂ *V* → *V*, *A*(*x*) = *x*(0)*A*, which allows us to rewrite system (5) in *V* as

(8) $$\begin{array}{c} \dot{v}\left(t\right)=A\left(\varphi_{0}\left(t,x\right)\right)v\left(t\right),\;t>0,\\ v\left(0\right)=v_{0}. \end{array}$$

The mapping

(9) $$\begin{array}{c} \Phi_{0}:{\mathbb{R}}_{+}\times X\to ℬ\left(V\right),\;\Phi_{0}\left(t,x\right)v=S\left(\int_{0}^{t}x\left(s\right)ds\right)v \end{array}$$

is a classic cocycle over the semiflow *φ*_0_, and *C*_0_ = (*φ*_0_, Φ_0_) is a linear skew-product semiflow strongly continuous on *Y*. Also, for all *v*_0_ ∈ *D*(*A*), we have obtained that *v*(*t*) = Φ(*t*, *x*)*x*_0_, *t* ≥ 0, is a strongly solution of system (8). As *C*_0_ = (*φ*_0_, Φ_0_) is a skew-product semiflow on *Y*, then the mapping *C* : *T* × *Y* → *Y*, *C*(*t*, *s*, *x*, *v*) = (*φ*(*t*, *s*, *x*), Φ(*t*, *s*, *x*)*v*), where

(10) φ(t,s,x)=φ0(t−s,x),  Φ(t,s,x)=Φ0(t−s,x),             ∀(t,s,x)∈T×X,

is a skew-evolution semiflow on *Y*. Hence, the skew-evolution semiflows generalize the notion of skew-product semiflows.

More directly, if Φ(*t*, *s*, *x*) is the solution of the Cauchy problem

(11) $$\begin{array}{c} v^{\prime }\left(t\right)=A\left(\varphi \left(t,s,x\right)\right)v\left(t\right),\;t>s,\\ v\left(s\right)=x, \end{array}$$

then *C* = (*φ*, Φ) is a linear skew-evolution semiflow.

Let us recall the definition of a semigroup of linear operators, and let us give an example which shows that this is generating a skew-evolution semiflow.

**Definition 7**

A mapping *S* : ℝ_+_ → *ℬ*(*V*) is called *semigroup* of linear operators on *V* if the following relations hold:

(sgr1) $$\begin{array}{c} S\left(0\right)=I, \end{array}$$

(sgr2) $$\begin{array}{c} S\left(t\right)S\left(s\right)=S\left(t+s\right),\;\forall \left(t,s\right)\in {\mathbb{R}}_{+}^{2}. \end{array}$$

**Example 8**

One can naturally associate to every semigroup of operators the mapping Φ_*S*_ : *T* × *X* → *ℬ*(*V*), defined by Φ_*S*_(*t*, *s*, *x*) = *S*(*t* − *s*), which is an evolution cocycle on *V* over evolution semiflows given, for example, by *φ*(*t*, *s*, *x*) = *x*_*t*−*s*_ (see Example 4).

Other examples of skew-evolution semiflows are given in [6]. The asymptotic properties, as well as their characterizations, are given by means of norms of the trajectories or orbits of *v*, given by *t* → Φ(*t*, *t*_0_, *x*)*v*, which are considered measurable. A particular case of skew-evolution semiflows is given by the following.

**Definition 9**

A skew-evolution semiflow *C* = (*φ*, Φ) is ∗*-strongly measurable* if, for every (*t*, *t*_0_, *x*, v*) ∈ *T* × *X* × V*, the mapping given by *s* ↦ ||Φ(*t*,*s*,*φ*(*s*,*t*_0_,*x*))*v*|| is measurable on [*t*_0_, *t*].

## 3. On Trichotomy Issues

We intend to give a new approach for the property of trichotomy for skew-evolution semiflows, the *ω*-trichotomy. Some examples and connections with the classic concept of exponential trichotomy are also provided.

Let *C* : *T* × *Y* → *Y*, *C*(*t*, *s*, *x*, *v*) = (*φ*(*t*, *s*, *x*), Φ(*t*, *s*, *x*)*v*) be a skew-evolution semiflow on *Y*. We recall that a mapping *P* : *X* → *ℬ*(*V*) with the property

(12) $$\begin{array}{c} P{\left(x\right)}^{2}=P\left(x\right),\;\forall x\in X \end{array}$$

is called *projections family* on *V*.

**Definition 10**

A projections family *P* : *X* → *ℬ*(*V*) is said to be *invariant* relative to the skew-evolution semiflow *C* = (*φ*, Φ) if

(13) Φ(t,s,x)P(x)=P(φ(t,s,x))Φ(t,s,x),           ∀(t,s,x)∈T×X.

The splitting of the state space into three subspaces will be assured by the following.

**Definition 11**

Three projections families {*P*_*k*_}_*k*∈{1,2,3}_ are said to be *compatible* with a skew-evolution semiflow *C* = (*φ*, Φ) if

(c_1_) each of *P*_*k*_, *k* ∈ {1,2, 3} is invariant relative to *C*;

(c_2_)  for all *x* ∈ *X*, the projections verify the relations

(14) P1(x)+P2(x)+P3(x)=I,  Pi(x)Pj(x)=0,              ∀i,j∈{1,2,3}  i≠j.

In what follows we introduce the elements which will allow us to introduce a new concept of trichotomy for skew-evolution semiflows. We consider a mapping *ψ* : ℝ_+_ → ℝ_+_*, and we define

(15) $$\begin{array}{c} A_{\psi }=\left\{\omega \in \mathbb{R},\;\;\underset{t\geq 0}{\sup}\;\psi \left(t\right)e^{-\omega t}<\infty \right\},\\ B_{\psi }=\left\{\omega \in \mathbb{R},\;\;\underset{t\geq 0}{\inf}\psi \left(t\right)e^{-\omega t}>0\right\}. \end{array}$$

**Remark 12**

For every function *ψ* : ℝ_+_ → ℝ_+_*, the following statements hold:

(i) *A*_*ψ*_ ≠ *∅* and *B*_*ψ*_ ≠ *∅*;
(ii) *μ* ∈ *A*_*ψ*_ implies the existence of a constant *M* ∈ ℝ_+_ such that
  (16) $$\begin{array}{c} \psi \left(t\right)\leq Me^{\mu t},\;\forall t\geq 0; \end{array}$$
(iii) *λ* ∈ *B*_*h*_ implies that there exists a constant *m* ∈ ℝ_+_ such that
  (17) $$\begin{array}{c} \psi \left(t\right)\geq me^{\lambda t},\;\forall t\geq 0. \end{array}$$

Let us denote that

(18) ω−ψ={inf Aψ,if  Aψ≠∅,∞,if  Aψ=∅,ω_ψ={sup⁡Bψ,if  Bψ≠∅,−∞,if  Bψ=∅.

**Definition 13**

A skew-evolution semiflow *C* = (*φ*, Φ) is called *ω-trichotomic* if there exist three projections families {*P*_*k*_}_*k*∈{1,2,3}_ compatible with *C* and some functions *ψ*, *ζ*, *χ* : ℝ_+_ → ℝ_+_* with the properties

(19) $$\begin{array}{c} {\overset{-}{\omega }}_{\psi }<0,\;{\underline{\omega }}_{\zeta }>0,\;{\bar{\omega }}_{\chi }<\infty ,\;{\underline{\omega }}_{\chi }<0, \end{array}$$

such that

(*t*_1_) ||Φ(*t*, *t*_0_, *x*)*P*_1_(*x*)*v*|| ≤ *ψ*(*t* − *t*_0_)||*P*_1_(*x*)*v*||;
(*t*_2_) *ζ*(*t* − *t*_0_)||*P*_2_(*x*)*v*|| ≤ ||Φ(*t*, *t*_0_, *x*)*P*_2_(*x*)*v*||;
(*t*_3_) ||Φ(*t*, *t*_0_, *x*)*P*_3_(*x*)*v*|| ≤ *χ*(*t* − *t*_0_)||*P*_3_(*x*)*v*|| and *χ*(*t* − *t*_0_)||Φ(*t*, *t*_0_, *x*)*P*_3_(*x*)*v*|| ≥ ||*P*_3_(*x*)*v*||,
 for all (*t*, *t*_0_) ∈ *T* and all (*x*, *v*) ∈ *Y*.

**Example 14**

Let *f* : ℝ_+_ → (0, *∞*) be a decreasing function with the property that there exists lim⁡_*t*→*∞*_⁡*f*(*t*) = *l* > 0. Let us denote that *λ* > *f*(0). Let us consider the Banach space *V* = ℝ^3^ with the norm ||(*v*_1_, *v*_2_, *v*_3_)|| = |*v*_1_| + |*v*_2_| + |*v*_3_|, *v* = (*v*_1_, *v*_2_, *v*_3_) ∈ *V*. The mapping

(20) Φ:T×X→ℬ(V),Φ(t,t0,x)v  =(e−λ(t−t0)+∫t0tx(τ−t0)dτv1,e∫t0tx(τ−t0)dτv2,    e−(t−t0)x(0)+∫t0tx(τ−t0)dτv3),

where *t* ≥ *t*_0_ ≥ 0, (*x*, *v*) ∈ *Y*, is an evolution cocycle over the evolution semiflow given in Example 4. We define the projections families *P*_1_, *P*_2_, *P*_3_ : *X* → *ℬ*(ℝ^3^) by *P*_1_(*x*)*v* = (*v*_1_, 0,0), *P*_2_(*x*)*v* = (0, *v*_2_, 0), *P*_3_(*x*)*v* = (0,0, *v*_3_), for all *x* ∈ *X* and all *v* = (*v*_1_, *v*_2_, *v*_3_) ∈ ℝ^3^. The following inequalities

(21) ||Φ(t,t0,x)P1(x)v||  ≤e[−λ+x(0)](t−s)||Φ(s,t0,x)P1(x)v||,||Φ(t,t0,x)P2(x)v||  ≥el(t−s)||Φ(s,t0,x)P2(x)v||,||Φ(t,t0,x)P3(x)v||  ≤e−x(0)(t−s)||Φ(s,t0,x)P3(x)v||,||Φ(t,t0,x)P3(x)v||  ≥e−x(0)(t−s)||Φ(s,t0,x)P3(x)v||

hold for all (*t*, *s*), (*s*, *t*_0_) ∈ *T* and all (*x*, *v*) ∈ *Y*. The mappings *ψ*, *ζ*, *χ* : ℝ_+_ → ℝ_+_*, defined by

(22) $$\begin{array}{c} \psi \left(u\right)=e^{[-\lambda +x(0)]u},\;\;\zeta \left(u\right)=e^{lu},\;\;\chi \left(u\right)=e^{-x(0)u} \end{array}$$

satisfy relations (19). For *s* = *t*_0_ and according to Definition 2, ((et_1_)) we obtain relations (i)–(iii) in Definition 13. Hence, *C* is *ω*-trichotomic.

**Remark 15**

For *P*_3_ = 0, the property of *ω*-dichotomy is obtained. If we consider *P*_2_ = *P*_3_ = 0, we obtain the property of *ω*-stability and for *P*_1_ = *P*_3_ = 0, the property of *ω*-instability.

In what follows, if *P*_*k*_ is a given projections family, we will denote that

(23) $$\begin{array}{c} \Phi_{k}\left(t,s,x\right)=\Phi \left(t,s,x\right)P_{k}\left(x\right), \end{array}$$

for every (*t*, *s*), (*s*, *t*_0_) ∈ *T* and *x* ∈ *X*. We remark that the following relations hold:

- Φ_*k*_(*t*, *t*, *x*) = *P*_*k*_(*x*), ∀(*t*, *x*) ∈ ℝ_+_ × *X*;
- Φ_*k*_(*t*, *s*, *φ*(*s*, *t*_0_, *x*))Φ_*k*_(*s*, *t*_0_, *x*) = Φ_*k*_(*t*, *t*_0_, *x*), ∀(*t*, *s*), (*s*, *t*_0_) ∈ *T*, ∀*x* ∈ *X*.

**Proposition 16**

A skew-evolution semiflow *C* = (*φ*, Φ) is *ω*-trichotomic if and only if there exist some constants *N*_1_, *N*_2_, *N*_3_ ≥ 1, *ν*_1_, *ν*_2_, *ν*_3_ > 0 and three projections families {*P*_*k*_}_*k*∈{1,2,3}_ compatible with *C* such that

(et1) $$\begin{array}{c} e^{\nu_{1}(t-t_{0})}||\Phi_{1}\left(t,t_{0},x\right)v||\leq N_{1}||P_{1}\left(x\right)v||, \end{array}$$

(et2) $$\begin{array}{c} e^{\nu_{2}(t-t_{0})}||P_{2}\left(x\right)v||\leq N_{2}||\Phi_{2}\left(t,t_{0},x\right)v||, \end{array}$$

(et3) ||P3(x)v||≤N3eν3(t−t0)||Φ3(t,t0,x)v||≤N32e2ν3(t−t0)||P3(x)v||,

for all (*t*, *t*_0_) ∈ *T* and all (*x*, *v*) ∈ *Y*.

**Proof**

*Necessity*. Let *C* be *ω*-trichotomic. Then there exist three projections families compatible with *C* and three functions *ψ*, *ζ*, *χ* : ℝ_+_ → ℝ_+_* with the properties

(24) $$\begin{array}{c} {\overset{-}{\omega }}_{\psi }<0,\;{\underline{\omega }}_{\zeta }>0,\;{\overset{-}{\omega }}_{\chi }<\infty ,\;{\underline{\omega }}_{\chi }<0, \end{array}$$

such that relations (i)–(iii) of Definition 13 are verified. For *μ* ∈ *A*_*ψ*_, there exists a constant *M* ∈ ℝ_+_ such that *ψ*(*t*) ≤ *Me* ^*μt*^, for all  *t* ≥ 0, and for *λ* ∈ *B*_*ψ*_ there exists a constant *m* ∈ ℝ_+_ with the property *ψ*(*t*) ≥ *me* ^*λt*^, for all *t* ≥ 0.

As ${\overset{-}{\omega }}_{\psi }<0$, there exist *N*_1_ ≥ 1 and *ν*_1_ > 0, such that *ψ*(*t*) ≤ *N*_1_ *e* ^−*ν*_1_*t*^, for all *t* ≥ 0. Hence, relation ((et_1_)) is obtained. As ${\underline{\omega }}_{\zeta }>0$, it follows that there exist *N*_2_ ≥ 1 and *ν*_2_ > 0, such that *ζ*(*t*) ≥ *N*_2_ *e* ^*ν*_2_*t*^, for all *t* ≥ 0, which implies ((et_2_)). From ${\overset{-}{\omega }}_{\chi }<\infty$ and ${\underline{\omega }}_{\chi }<0$, it follows that there exist *N*_3_ ≥ 1, *ν*_3_ > 0 with the property

(25) $$\begin{array}{c} \frac{1}{N_{3}}e^{-\nu_{3}t}\leq \chi \left(t\right)\leq N_{3}e^{\nu_{3}t},\;\forall t\geq 0. \end{array}$$

Hence, relation ((et_3_)) is satisfied.

*Sufficiency*. Let us assume that there exist three projections families compatible with *C* and *N*_1_, *N*_2_, *N*_3_ ≥ 1, *ν*_1_, *ν*_2_, *ν*_3_ > 0 such that relations ((et_1_))–((et_3_)) hold. Let us define the mappings *ψ*, *ζ*, *χ* : ℝ_+_ → ℝ_+_* by

(26) $$\begin{array}{c} \psi \left(t\right)=N_{1}e^{-\nu_{1}t},\;\;\zeta \left(t\right)=N_{2}^{-1}e^{\nu_{2}t},\;\;\chi \left(t\right)=N_{3}e^{\nu_{3}t}. \end{array}$$

We obtain the relations

(27) $$\begin{array}{c} {\overset{-}{\omega }}_{\psi }<0,\;{\underline{\omega }}_{\zeta }>0,\;{\overset{-}{\omega }}_{\chi }<\infty ,\;{\underline{\omega }}_{\chi }<0. \end{array}$$

Hence, relations (i)–(iii) of Definition 13 are verified, and, therefore, *C* is *ω*-trichotomic, which ends the proof.

**Remark 17**

Proposition 16 is in fact the classic definition of exponential trichotomy. On the other hand, in Definition 13, the exponentials are not implied.

## 4. Main Results

We obtain a characterization for the property of trichotomy, by means of the shifted skew-evolution semiflow.

**Theorem 18**

A skew-evolution semiflow *C* = (*φ*, Φ) is *ω*-trichotomic if and only if there exist three projections families {*P*_*k*_}_*k*∈{1,2,3}_ compatible with *C* and

- the evolution cocycle Φ_1_ is exponentially stable;
- the evolution cocycle Φ_2_ is exponentially instable;
- there exists a constant *λ* > 0 such that the *λ*-shifted evolution cocycle Φ_*λ*_^3^ is exponentially stable and the −*λ*-shifted evolution cocycle Φ_−*λ*_^3^ is exponentially instable.

**Proof**

*Necessity.* Statements (i) and (ii) are obtained immediately from the necessity of Proposition 16. According to ((et_3_)), there exist *N*_3_ ≥ 1 and *ν*_3_ > 0 such that

(28) ||Φ3(t,t0,x)v||≤N3eν3(t−t0)||P3(x)v||,     ∀(t,t0)∈T,  ∀(x,v)∈Y.

Let us consider that *λ* = 2*ν*_3_ > 0. We obtain successively

(29) ||Φλ3(t,t0,x)v||=e−λ(t−t0)||Φ3(t,t0,x)v||≤N3e−λ(t−t0)eν3(t−t0)||P3(x)v||=N3e−ν3(t−t0)||P3(x)v||,

for all (*t*, *t*_0_) ∈ *T* and all (*x*, *v*) ∈ *Y*, which shows that Φ_*λ*_^3^ is exponentially stable. Also, we have

(30) e−ν3(t−t0)||P3(x)v||≤N3||Φ3(t,t0,x)v||,        ∀(t,t0)∈T,  ∀(x,v)∈Y.

If we consider that *λ* = 2*ν*_3_ > 0, we obtain, according also to Definition 2  ((ec_2_)),

(31) ||Φ−λ3(s,t0,x)v||=eλ(s−t0)||Φ3(s,t0,x)v||≤N3eν3(t−s)eλ(s−t0)||Φ3(t,t0,x)v||=N3e−ν3(t−s)||Φ−λ3(t,t0,x)v||,

for all (*t*, *s*), (*s*, *t*_0_) ∈ *T* and all (*x*, *v*) ∈ *Y*, which proves that Φ_−*λ*_^3^ is exponentially instable.

*Sufficiency*. Relations (i) and (ii) in Definition 13 are obtained from the sufficiency of Proposition 16. As there exists a constant *λ* > 0 such that the skew-evolution semiflow Φ_*λ*_^3^ is exponentially stable, then there exists some constants *N* ≥ 1 and *ν* > 0 such that

(32) ||Φλ3(t,t0,x)v||≤Ne−ν(t−t0)||P3(x)v||,        ∀t≥t0≥0, ∀(x,v)∈Y.

Further, we obtain

(33) ||Φ3(t,t0,x)v||=eλ(t−t0)||Φλ3(t,t0,x)v||≤Neλ(t−t0)e−ν(t−s)||Φλ3(s,t0,x)v||=Neλ(t−s)e−ν(t−s)||Φ3(s,t0,x)v||,

for all (*t*, *s*), (*s*, *t*_0_) ∈ *T* and all (*x*, *v*) ∈ *Y*. Denoting

(34) $$\begin{array}{c} \nu_{3}=\{\begin{array}{cc} \lambda -\nu , & \text{if }\lambda >\nu ,\\ 1, & \text{if }\lambda \leq \nu , \end{array} \end{array}$$

the first relation in ((et_3_)) is obtained.

If there exists a constant *λ* > 0 such that the −*λ*-shifted skew-evolution semiflow *C*_−*λ*_ is exponentially instable, then there exist some constants *N* ≥ 1 and *ν* > 0 such that

(35) ||Φ(s,t0,x)v||=e−λ(s−t0)||Φ−λ(s,t0,x)v||≤Ne−ν(t−s)e−λ(s−t0)||Φ−λ(t,t0,x)v||=Ne−ν(t−s)eλ(t−s)||Φ(t,t0,x)v||,

for all (*t*, *s*), (*s*, *t*_0_) ∈ *T* and all (*x*, *v*) ∈ *Y*. If we consider *ν*_3_ defined as in (34), the second relation in ((et_3_)) is obtained. Hence, also (iii) in Definition 13 is true and *C* is *ω*-trichotomic.

Another characterization for the property of *ω*-trichotomy is given relative to the dual space V* of the Banach space *V*. To this aim, let us consider three projections families {*P*_*k*_}_*k*∈{1,2,3}_ compatible with *C* such that the evolution cocycle Φ_1_ has exponential growth and the evolution cocycle Φ_2_ has exponential decay.

**Theorem 19**

A ∗-*strongly measurable* skew-evolution semiflow *C* = (*φ*, Φ) is *ω*-trichotomic if the and only if the following statements hold:

(i) there exists $\tilde{N}\geq 1$ such that
  (36) $$\begin{array}{c} \int_{t_{0}}^{t}{||\Phi_{1}{\left(t,s,\varphi \left(s,t_{0},x\right)\right)}^{*}v^{*}||}^{p}ds\leq \tilde{N}{||P_{1}\left(x\right)v^{*}||}^{p}, \end{array}$$
  for all *p* > 1, all (*t*, *t*_0_) ∈ *T*, and all (*x*, v*) ∈ *X* × V*;
(ii) there exists $\bar{N}\geq 1$ such that
  (37) $$\begin{array}{c} {\left(\int_{t_{0}}^{t}{||\Phi_{2}\left(s,t_{0},x\right)v||}^{p}ds\right)}^{1/p}\leq \bar{N}||\Phi_{2}\left(t,t_{0},x\right)v||, \end{array}$$
  for all *p* > 1, all (*t*, *t*_0_) ∈ *T*, and all (*x*, *v*) ∈ *Y*;
(iii) there exist *α* < 0 and $\tilde{M}\geq 1$ such that
  (38) $$\begin{array}{c} \int_{t_{0}}^{\infty }e^{\alpha (\tau -t_{0})}||\Phi_{3}\left(\tau ,t_{0},x\right)v||d\tau \leq \tilde{M}||P_{3}\left(x\right)v||, \end{array}$$
  for all *t*_0_ ∈ ℝ_+_ and all (*x*, *v*) ∈ *Y* and there exist *β* < 0 and $\overset{-}{M}\geq 1$ such that
  (39) $$\begin{array}{c} \int_{t_{0}}^{t}e^{\beta (t-\tau )}||\Phi_{3}\left(\tau ,t_{0},x\right)v||d\tau \leq \bar{M}||\Phi_{3}\left(t,t_{0},x\right)v||, \end{array}$$
  for all (*t*, *t*_0_) ∈ *T* and all (*x*, *v*) ∈ *Y*.

**Proof**

*Necessity. * (i) As *C* is *ω*-trichotomic, according to Definition 13, there exists a mapping *ψ* : ℝ_+_ → ℝ_+_*, with the property ${\bar{\omega }}_{\psi }<0$, such that

(40) ||Φ(t,t0,x)P1(x)v||≤ψ(t−t0)||P1(x)v||,           ∀(t,t0)∈T,  (x,v)∈Y.

An equivalent relation is obtained, if we consider Definition 2  ((ec_2_)), given by

(41) ||Φ1(t,t0,x)v||=||Φ1(t,s,φ(s,t0,x))Φ1(s,t0,x)v||≤ψ(t−s)||Φ1(s,t0,x)v||,

for all (*t*, *t*_0_) ∈ *T* and all (*x*, *v*) ∈ *Y*. According to the properties of function *ψ*, there exist *N* ≥ 1 and *ν* > 0 such that the following relations hold:

(42) ∫t0t||Φ1(t,s,φ(s,t0,x))∗v∗||pds  ≤∫t0tψp(t−s)||Φ1(s,s,φ(s,t0,x))∗v∗||pds  ≤Np∫t0te−pν(t−s)||P1(x)v∗||pds  ≤N~||P1(x)v∗||p,

for all (*t*, *t*_0_) ∈ *T* and all (*x*, v*) ∈ *X* × V*, where we have denoted that $\tilde{N}=N^{p}/\nu p$.

(ii) According to Definition 13, there exists a mapping *ζ* : ℝ_+_ → ℝ_+_*, with the property ${\underline{\omega }}_{\zeta }<0$, such that

(43) ζ(t−t0)||P2(x)v||≤||Φ2(t,t0,x)v||,       ∀(t,t0)∈T,  (x,v)∈Y.

The property of function *ζ* assures the existence of some constants *N* ≥ 1 and *ν* > 0, such that, for all *p* > 0, the following relations hold:

(44) (∫t0t||Φ2(s,t0,x)v||pds)1/p≤N||Φ2(t,t0,x)v|| ×(∫t0te−νp(t−s)ds)1/p≤N¯||Φ2(t,t0,x)v||,

for all (*t*, *t*_0_) ∈ *T* and all (*x*, *v*) ∈ *Y*, where we have denoted that $\overset{-}{N}=N/{\left(\nu p\right)}^{1/p}$.

(iii) Both relations are obtained by a similar proof as in (i) and (ii), according to Theorem 18.

*Sufficiency. * (i) As the evolution cocycle Φ_1_ has exponential growth, there exist *M* ≥ 1 and *ρ* > 0 such that

(45) ||Φ1(t,t0,x)v||≤Meρ(t−s)||Φ1(s,t0,x)v||,     ∀(t,s),(s,t0)∈T,  (x,v)∈Y.

Let *t* ≥ *t*_0_ + 1. We consider that

(46) $$\begin{array}{c} \int_{t_{0}}^{t_{0}+1}e^{-\rho (s-t_{0})}ds=\int_{0}^{1}e^{-\rho \tau }d\tau =\frac{1-e^{-\rho }}{\rho }=c>0. \end{array}$$

For every *p* > 1 there exists *q* > 1 such that 1/*p* + 1/*q* = 1. We obtain

(47) c|〈v∗,Φ1(t,t0,x)v〉|  ≤∫t0t0+1e−ρ(s−t0)|〈v∗,Φ1(t,t0,x)v〉|ds  ≤∫t0t0+1e−ρ(s−t0)||Φ1(t,s,φ(s,t0,x))∗v∗||     ×||Φ1(s,t0,x)v||ds  ≤M||v||∫t0t0+1||Φ1(t,s,φ(s,t0,x))∗v∗||ds  ≤(∫t0t0+1||Φ1(t,s,φ(s,t0,x))∗v∗||pds)1/p   ×(∫t0t0+1Mq||P1(x)v||qds)1/q  ≤M||v||(∫t0t||Φ1(t,s,φ(s,t0,x))∗v∗||pds)1/p  ≤MN~||P1(x)v||||P1(x)v∗||.

Hence, we have that

(48) ||Φ1(t,s,φ(s,t0,x))v||≤MN~c||P1(x)v||,       ∀t≥t0+1,  ∀(x,v)∈Y.

For *t* ∈ [*t*_0_, *t*_0_ + 1] we obtain

(49) $$\begin{array}{c} ||\Phi_{1}\left(t,s,\varphi \left(s,t_{0},x\right)\right)v||\leq Me^{\rho }||P_{1}\left(x\right)v||,\;\forall \left(x,v\right)\in Y, \end{array}$$

which implies that

(50) ||Φ1(t,s,φ(s,t0,x))v||≤M1||P1(x)v||,      ∀(t,t0)∈T,  (x,v)∈Y,

where we have denoted

(51) $$\begin{array}{c} M_{1}=M\left(e^{\rho }+\frac{\tilde{N}}{c}\right). \end{array}$$

Further, the following relations hold:

(52) |〈v∗,Φ1(t,t0,x)v〉|  =|〈v∗,Φ1(t,s,φ(s,t0,x))Φ1(s,t0,x)v〉|  =|〈Φ1(t,s,φ(s,t0,x))∗v∗,Φ1(s,t0,x)v〉|  ≤M1||P1(x)v||||Φ1(t,s,φ(s,t0,x))∗v∗||.

By integrating on [*t*_0_, *t*], it follows that

(53) (t−t0)|〈v∗,Φ1(t,t0,x)v〉|  ≤M1||P1(x)v||∫t0t||Φ1(t,s,φ(s,t0,x))∗v∗||ds  ≤M1N~||P1(x)v||||P1(x)v∗||,

and further

(54) $$\begin{array}{c} \left(t-t_{0}\right)||\Phi_{1}\left(t,t_{0},x\right)v||\leq M_{1}\tilde{N}||P_{1}\left(x\right)v||, \end{array}$$

which implies that

(55) ||Φ1(t,t0,x)v||≤ψ(t−t0)||P1(x)v||,      ∀(t,t0)∈T,  (x,v)∈Y,

where we have considered $\psi \left(u\right)=M_{1}\left(\tilde{N}+1\right)/u$, which satisfies ${\overset{-}{\omega }}_{\psi }<0$. Statement (*t*_1_) of Definition 13 is hence obtained.

(ii) Let (*t*, *t*_0_) ∈ *T*. Let us denote that

(56) $$\begin{array}{c} F\left(t\right)=\int_{t_{0}}^{t}{||\Phi_{2}\left(s,t_{0},x\right)v||}^{p}ds. \end{array}$$

According to the fact that $F\left(t\right)\leq {\bar{N}}^{p}F^{\prime }\left(t\right)$, for all *t* ≥ 0, we obtain, for all (*x*, *v*) ∈ *Y*,

(57) $$\begin{array}{c} F\left(t_{0}+1\right)e^{(t-t_{0}-1)/{\bar{N}}^{p}}\leq F\left(t\right)\leq {\bar{N}}^{p}{||\Phi_{2}\left(t,t_{0},x\right)v||}^{p}. \end{array}$$

As the evolution cocycle Φ_2_ has exponential decay, there exist some constants *M* ≥ 1 and *ρ* > 0 such that

(58) ||Φ2(s,t0,x)v||≤Meρ(t−s)||Φ2(t,t0,x)v||,     ∀(t,s),(s,t0)∈T,  (x,v)∈Y.

Let *s* ∈ [*t*_0_, *t*_0_ + 1). We obtain

(59) $$\begin{array}{c} {||P_{2}\left(x\right)v||}^{p}\leq M^{p}e^{p\rho (t-s)}\int_{t_{0}}^{t_{0}+1}{||\Phi_{2}\left(s,t_{0},x\right)v||}^{p}ds \end{array}$$

and further

(60) $$\begin{array}{c} M^{-p}e^{-p\rho (t-s)}{||P_{2}\left(x\right)v||}^{p}\leq F\left(t_{0}+1\right). \end{array}$$

For *t* ≥ *t*_0_ + 1, we obtain

(61) $$\begin{array}{c} \frac{1}{M^{p}e^{\rho p}}e^{-1/{\bar{N}}^{p}}e^{(t-t_{0})/{\bar{N}}^{p}}{||v||}^{p}\leq {\bar{N}}^{p}{||\Phi_{2}\left(t,t_{0},x\right)v||}^{p}, \end{array}$$

and, if *t* ∈ [*t*_0_, *t*_0_ + 1],

(62) $$\begin{array}{c} ||P_{2}\left(x\right)v||\leq Me^{\rho }e^{1/{p^{\overset{-}{N}}}^{p}}e^{\left(-1/{p^{\overset{-}{N}}}^{p})(t-t_{0}\right)}||\Phi_{2}\left(t,t_{0},x\right)v||, \end{array}$$

for all (*x*, *v*) ∈ *Y*. Hence,

(63) ζ(t−t0)||P2(x)v||≤||Φ2(t,t0,x)v||,     ∀(t,t0)∈T,  (x,v)∈Y,

where we have considered *ζ* : ℝ_+_ → ℝ_+_* defined by

(64) $$\begin{array}{c} \zeta \left(u\right)=\frac{1}{\bar{N}Me^{\rho }}e^{-1/{p^{\overset{-}{N}}}^{p}}e^{(1/{p^{\overset{-}{N}}}^{p})u}, \end{array}$$

with the property ${\underline{\omega }}_{\zeta }>0$. Statement (*t*_2_) of Definition 13 is proved.

(iii) A similar proof for the *α*-shifted evolution cocycle Φ_*α*_^3^ in (i) and for the *β*-shifted evolution cocycle Φ_*β*_^3^ in (ii) leads to the existence of two functions *χ*_1_, *χ*_2_ : ℝ_+_ → ℝ_+_*, with the properties ${\bar{\omega }}_{\chi_{1}}<0$ and ${\underline{\omega }}_{\chi_{2}}>0$, such that

(65) ||Φα3(t,t0,x)v||≤χ1(t−t0)||P3(x)v||,      ∀(t,t0)∈T,  (x,v)∈Y,χ2(t−t0)||P3(x)v||≤||Φβ3(t,t0,x)v||,      ∀(t,t0)∈T,  (x,v)∈Y.

If we consider the definition of the shifted evolution cocycle, the previous relations are equivalent with

(66) ||Φ3(t,t0,x)v||≤e−α(t−t0)χ1(t−t0)||P3(x)v||,         ∀(t,t0)∈T,  (x,v)∈Y,e−β(t−t0)χ2(t−t0)||P3(x)v||≤||Φ3(t,t0,x)v||,         ∀(t,t0)∈T,  (x,v)∈Y.

We define $\tilde{\chi },\bar{\chi }:{\mathbb{R}}_{+}\to {\mathbb{R}}_{+}^{*}$ by $\tilde{\chi }\left(u\right)=e^{-\alpha u}\chi_{1}\left(u\right)$ and $\bar{\chi }\left(u\right)=e^{-\beta u}\chi_{2}\left(u\right)$, which have the properties ${\bar{\omega }}_{\tilde{\chi }}<\infty$ and ${\underline{\omega }}_{\bar{\chi }}<0$. Hence, property (*t*_3_) of Definition 13 is obtained.

**Remark 20**

The property described by relation (36) is also called ∗-strong integral stability for a skew-evolution semiflow. Relation (36) is a characterization of Barbashin type in the strong topology, and relations (37), (38), and (39) are characterizations of Datko type for the asymptotic properties of skew-evolution semiflows involved in the definition of *ω*-trichotomy.
